# Hemoglobin–Albumin–Lymphocyte–Platelet (HALP) Score as a Novel Biomarker for Predicting Coronary Slow Flow in Patients with Angina and/or Ischemia and Nonobstructive Coronary Arteries

**DOI:** 10.3390/jcm15031302

**Published:** 2026-02-06

**Authors:** Çağatay Tunca, Reha Yasin Şengül, Mehmet Taha Özkan, Alperen Taş, Yusuf Bozkurt Şahin, Saadet Demirtaş İnci, Veysel Ozan Tanık, Bülent Özlek

**Affiliations:** 1 Department of Cardiology, Ankara Etlik City Hospital, Ministry of Health, Ankara 06170, Türkiye; rehasengul@gmail.com (R.Y.Ş.); mehmettahaozkan@gmail.com (M.T.Ö.); ybozkurtsahin@gmail.com (Y.B.Ş.); saadet_demirtas@yahoo.com (S.D.İ.); drozantanik@gmail.com (V.O.T.); 2 Department of Cardiology, Ahi Evran University, Training and Research Hospital, Kırşehir 40100, Türkiye; alperentas555@hotmail.com; 3 Department of Cardiology, School of Medicine, Muğla Sıtkı Koçman University, Muğla 48000, Türkiye; bulent_ozlek@hotmail.com

**Keywords:** angina with non-obstructive coronary arteries, coronary slow flow phenomenon, HALP score, inflammation, microvascular dysfunction

## Abstract

**Background:** The coronary slow flow phenomenon (CSFP) is an angiographic entity increasingly recognized in patients with angina and/or ischemia but non-obstructive coronary arteries (ANOCA/INOCA), associated with systemic inflammation, endothelial dysfunction, and microvascular abnormalities. The hemoglobin, albumin, lymphocyte, and platelet (HALP) score is a novel immunonutritional index that may reflect this multifactorial risk profile. **Methods:** This retrospective single-center case–control study included 122 patients with CSFP and 126 age- and sex-matched controls with normal coronary flow, all presenting with symptoms of chronic coronary syndrome. CSFP was diagnosed via corrected TIMI frame count. HALP and other inflammatory indices (NLR, PLR, SII, SIRI) were calculated from baseline laboratory values. Associations were evaluated using multivariable logistic regression, ROC analysis, and restricted cubic spline (RCS) modeling. **Results:** The HALP score was significantly lower in CSFP patients (mean 56.2 vs. 65.9, *p* < 0.001). In multivariable analysis, HALP was independently associated with CSFP (adjusted OR: 0.951; 95% CI: 0.930–0.972; *p* < 0.001), whereas NLR lost significance. PLR, SII, and SIRI remained independently associated. HALP showed the highest diagnostic performance (AUC: 0.698), significantly outperforming all other indices (DeLong *p* < 0.001). A HALP cutoff ≤ 56.4 provided 58.2% sensitivity and 77.0% specificity. RCS analysis demonstrated a significant non-linear inverse relationship (*p* for non-linearity = 0.034). Subgroup analyses confirmed consistent associations across age, sex, hypertension, and diabetes strata. **Conclusions:** The HALP score is independently associated with CSFP and outperforms traditional inflammatory indices. Its low cost and accessibility make it a promising tool for clinical risk stratification in ANOCA/INOCA patients, pending validation in multicenter prospective studies.

## 1. Introduction

The coronary slow flow phenomenon (CSFP) is an angiographic finding characterized by delayed opacification of the epicardial coronary arteries despite the absence of obstructive coronary lesions [1]. Once considered a benign or incidental angiographic variant, CSFP is now increasingly recognized as a clinically relevant entity associated with recurrent chest pain, effort-induced angina, arrhythmias, and even acute coronary syndromes (ACS) [2]. It has been reported in approximately 1% to 7% of patients undergoing coronary angiography (CAG) and often presents in individuals with angina and angiographically normal or near-normal coronary arteries [3].

This clinical profile places CSFP within the broader spectrum of ischemia and/or angina with non-obstructive coronary arteries (INOCA/ANOCA), a heterogeneous group encompassing mechanisms of myocardial ischemia beyond epicardial obstruction [4]. Among these, coronary microvascular dysfunction (CMD) is emerging as a central pathophysiological substrate. CSFP, owing to its angiographic visibility, may reflect diffuse microvascular and endothelial abnormalities in this population and thus represents a unique window into the systemic processes underlying ANOCA/INOCA [5]. Indeed, studies have shown that patients with CSFP often display markers of microvascular dysfunction, increased coronary resistance, and impaired coronary flow reserve, features that parallel those seen in CMD and small-vessel disease [6].

The pathogenesis of CSFP is complex and multifactorial, involving endothelial dysfunction, chronic low-grade inflammation, oxidative stress, autonomic imbalance, platelet activation, and metabolic dysregulation [6]. These mechanisms are interrelated and may act synergistically to impair coronary vasomotion, resulting in the characteristic slow flow observed during angiography [7]. Importantly, many of these processes are also implicated in systemic inflammatory states and immune-nutritional imbalance, further supporting the notion that CSFP may be a manifestation of broader vascular pathology [8].

In recent years, several hematological and inflammatory biomarkers have been investigated for their potential association with CSFP, including the neutrophil-to-lymphocyte ratio (NLR), platelet-to-lymphocyte ratio (PLR), systemic immune-inflammation index (SII), and systemic inflammation response index (SIRI) [9,10,11]. Although these indices provide valuable insights into systemic inflammation, they often capture only a single dimension of the underlying pathophysiology. In contrast, the hemoglobin, albumin, lymphocyte, and platelet (HALP) score is a novel composite index that integrates nutritional status, immune function, and inflammatory burden [12]. However, its role in microvascular coronary disorders such as CSFP remains unexplored.

Given the systemic nature of the processes implicated in CSFP and the need for simple, cost-effective markers to improve risk stratification in ANOCA/INOCA populations, we hypothesized that the HALP score could serve as a clinically useful biomarker in this setting. Therefore, the present study aimed to investigate the association between HALP and CSFP in patients with ANOCA/INOCA and to compare the diagnostic performance of HALP with that of other established inflammatory indices.

## 2. Materials and Methods

### 2.1. Study Population

This retrospective case–control study included symptomatic patients who underwent elective invasive coronary angiography (CAG) between October 2022 and September 2023 for suspected chronic coronary syndrome (CCS), as defined by the European Society of Cardiology (ESC) guidelines [13]. All patients were evaluated by a cardiologist for chronic ischemic heart disease and fulfilled the diagnostic criteria for CCS based on clinical symptoms suggestive of myocardial ischemia in conjunction with objective evidence of ischemia or coronary artery disease. Patients were diagnosed with CCS if at least one of the following criteria was present: (i) coronary lesions detected on elective non-invasive coronary imaging, such as coronary computed tomography angiography; (ii) positive findings for myocardial ischemia on exercise stress testing; or (iii) evidence of myocardial ischemia on myocardial perfusion scintigraphy. Patients in whom subsequent elective invasive CAG demonstrated non-obstructive coronary artery disease were eligible for inclusion.

A total of 2311 patients were initially screened. Among these, 122 patients diagnosed with CSFP constituted the CSFP group. For each patient with CSFP, two age- and sex-matched individuals with normal coronary flow were selected as controls (n = 126) using simple random sampling when more than two eligible controls were available.

**Figure 1:**
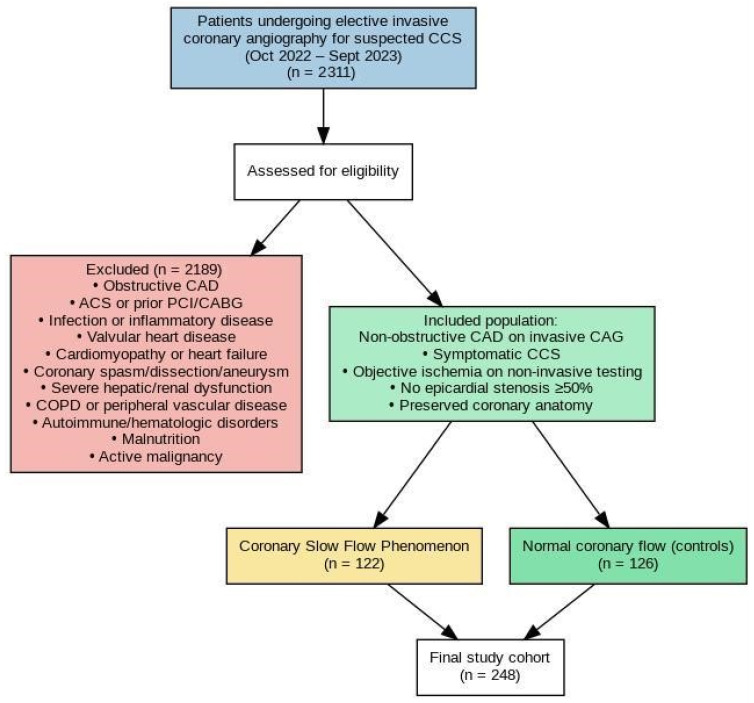
Flowchart of patient selection and study population. Abbreviations: ACS, Acute Coronary Syndrome; CABG, Coronary Artery By-pass Grafting; CAG, Coronary Angiography; CAD, Coronary Artery Disease; CCS, Chronic Coronary Syndrome; COPD, Chronic Obstructive Pulmonary Disease; PCI, Percutaneous Coronary Intervention.

A flowchart illustrating patient selection and exclusion criteria is presented in Figure 1. Exclusion criteria included a history of ACS; prior percutaneous coronary intervention (PCI) or coronary artery bypass grafting; acute or chronic infection; moderate-to-severe valvular heart disease; cardiomyopathy; HF; coronary artery aneurysm; coronary artery spasm or dissection; severe hepatic or renal dysfunction; chronic obstructive pulmonary disease; peripheral vascular disease; autoimmune disease; hematologic disorders; malnutrition; and active malignancy.

### 2.2. CAG and Thrombolysis in Myocardial Infarction (TIMI) Frame Count

All CAG procedures were performed using a General Electric Innova 2100 IQ system (GE Healthcare, Chicago, IL, USA) with the standard Judkins technique. Angiographic images were acquired in multiple projections, including right- and left-oblique views with cranial and caudal angulations, to optimize coronary visualization. Images were recorded at 30 frames per second. A nonionic, low-osmolar contrast agent (Iohexol, Omnipol 300 mg I/mL; Polifarma, Istanbul, Turkey) was used in all procedures.

CSFP was diagnosed using the TIMI frame count (TFC) method, as originally described by Gibson et al. [14]. The TFC represents the number of cine frames required for contrast to reach predefined distal landmarks in each major epicardial coronary artery. The first frame was defined as the frame in which contrast filled more than 70% of the arterial lumen, while the final frame corresponded to specific anatomical landmarks: the “whale’s tail” for the left anterior descending (LAD) artery, the most distal bifurcation of the obtuse marginal branch for the circumflex (LCx) artery, and the first posterolateral branch for the right coronary artery (RCA).

TFC measurements were obtained in the right anterior oblique–caudal projection for the LAD and LCx arteries, and in the left anterior oblique–cranial projection for the RCA. Because of the longer length of the LAD artery, raw LAD frame counts were divided by 1.7 to calculate the corrected TFC, with a normal reference value of 21.1 ± 1.5 frames. Normal reference values were defined as 22.1 ± 4.1 frames for the LCx artery and 20.4 ± 3.1 frames for the RCA. The mean TFC was calculated as the average frame count of the three major epicardial arteries. CSFP was diagnosed when the TFC of any coronary artery exceeded two standard deviations above the established normal reference values [15].

Representative angiographic videos of a patient with CSFP and a control subject with normal coronary flow are provided as Supplementary Material to illustrate the angiographic differences.

### 2.3. Laboratory Measurements

Venous blood samples were obtained from the antecubital vein after overnight fasting and prior to CAG. All routine hematological, biochemical, and lipid parameters were analyzed in the hospital’s central laboratory. Blood samples were collected in standardized EDTA-containing tubes and analyzed within two hours of collection. Hematological parameters were measured using an XT-2000i automated hematology analyzer (Sysmex Corp., Long Grove, IL, USA).

The HALP score was calculated using the following formula [12]:

HALP score = hemoglobin (g/L) × albumin (g/L) × lymphocyte count (/L) ÷ platelet count (/L)

The SII was calculated as follows [9]:

SII = (platelet count × neutrophil count)/lymphocyte count

The SIRI was calculated using the formula [11]:

SIRI = (neutrophil count × monocyte count)/lymphocyte count

All indices were calculated using baseline laboratory values obtained at admission prior to CAG. Follow-up or post-procedural laboratory measurements were not included in the analysis.

### 2.4. Ethical Considerations

This study was conducted in accordance with the Declaration of Helsinki and approved by the institutional ethics committee of Ankara Etlik City Hospital, Clinical Research Ethics Committee (Approval Date: 18 October 2023; Approval No: AESH-EKİ-2023-611). Given the study’s retrospective design and the use of anonymized data, the ethics committee waived the requirement for informed consent. All patient data were handled with strict confidentiality, and no identifiable personal information was used or disclosed.

### 2.5. Statistical Analysis

Continuous variables were tested for normality using visual inspection of histograms and the Kolmogorov–Smirnov test. Normally distributed continuous variables were presented as mean ± standard deviation and compared using the independent-samples *t*-test, whereas non-normally distributed variables were expressed as median (interquartile range) and compared using the Mann–Whitney *U* test. Categorical variables are presented as counts and percentages and were compared using the chi-square test or Fisher’s exact test, as appropriate. Univariable logistic regression analyses were initially performed to identify clinical and laboratory parameters associated with CSFP presence. Variables demonstrating a *p*-value < 0.10 in univariable analyses, as well as clinically relevant covariates, were considered for multivariable modeling. Given the substantial overlap in the biological components of inflammatory indices—including hematologic, immune, and inflammatory parameters—and the high potential for multicollinearity, multiple multivariable logistic regression models were constructed using a predefined modeling strategy. A core set of clinical covariates (age, sex, diabetes mellitus [DM], high-density lipoprotein [HDL] cholesterol, and C-reactive protein [CRP]) was included in all models. Each inflammatory index (HALP score, NLR, PLR, SII, and SIRI) was then introduced separately into individual models to allow an unbiased comparison of their independent associations with CSFP while avoiding collinearity-related distortion of effect estimates. The discriminative performance of the HALP score and other inflammatory indices for predicting CSFP was assessed using receiver operating characteristic (ROC) curve analysis. Areas under the ROC curve (AUCs) were calculated with corresponding 95% confidence intervals (CIs). The optimal cut-off value for the HALP score was determined using the Youden index. Sensitivity, specificity, positive predictive value (PPV), and negative predictive value (NPV) were subsequently calculated at this threshold. Pairwise comparisons of AUCs between the HALP score and the other inflammatory indices were performed using the DeLong test to statistically evaluate differences in discriminative performance. To explore potential non-linear associations between HALP score and the risk of CSFP, restricted cubic spline (RCS) analyses were conducted with four knots placed at default percentiles of the HALP distribution. Odds ratios (ORs) were plotted relative to the median HALP value. Non-linearity was formally tested by comparing the spline model with a linear model using the likelihood ratio test. Predefined subgroup analyses were performed to assess the consistency of the association between HALP score and CSFP across clinically relevant strata, including age (below vs. above the median), sex, hypertension status, and DM. Within each subgroup, adjusted odds ratios and 95% CIs were estimated using multivariable logistic regression models. Results were visually summarized using forest plots. Given the reduced sample sizes in certain strata, subgroup analyses were interpreted cautiously and treated as exploratory. All statistical analyses were performed using IBM SPSS Statistics version 26.0 (IBM Corp., Armonk, NY, USA). Graphical visualizations, including ROC curves, bar plots, RCS curves, and forest plots, were generated in Python version 3.11.2. A two-sided *p*-value < 0.05 was considered statistically significant.

## 3. Results

The baseline demographic, clinical, and laboratory characteristics of the study population are summarized in Table 1. There was no significant difference in age between the CSFP and normal flow groups. However, the proportion of male patients was significantly higher in the normal group (*p* = 0.011). The prevalence of hypertension, DM, and smoking did not differ significantly between the groups. Regarding medication use, the distribution of β-blockers, angiotensin-converting enzyme inhibitors, angiotensin II receptor blockers, and statins was comparable between the two groups. In laboratory findings, the CSFP group had significantly higher platelet counts and hemoglobin levels and lower serum albumin levels than the normal group (all *p* < 0.05). White blood cell, neutrophil, lymphocyte, and serum creatinine levels were comparable between the two groups. However, monocyte counts were significantly elevated in the CSFP group. Total cholesterol, triglycerides, and HDL-cholesterol levels showed no statistically significant differences. CRP levels were significantly higher in the CSFP group (*p* = 0.030). Among inflammatory indices, the HALP score was significantly lower in the CSFP group (56.16 ± 14.07 vs. 65.89 ± 14.56, *p* < 0.001), whereas NLR, PLR, SII, and SIRI were significantly higher (all *p* < 0.05). As expected, TFC for all three major coronary arteries was markedly elevated in the CSFP group, and the mean TFC was also significantly higher.

Univariable logistic regression analyses were performed to identify clinical and laboratory parameters associated with CSFP. As summarized in Table 2, male sex was significantly associated with CSFP (OR 1.917, 95% CI 1.158–3.176, *p* = 0.011). DM showed a trend toward significance (OR = 1.753, 95% CI, 0.953–3.223, *p* = 0.071), whereas age and hypertension were not significantly associated with CSFP.

Among laboratory variables, higher CRP levels were significantly associated with an increased likelihood of CSFP (OR 1.085, 95% CI 1.019–1.156, *p* = 0.011). Total cholesterol was not significantly associated with CSFP, while lower HDL-cholesterol levels demonstrated a borderline association (OR 0.957, 95% CI 0.912–1.004, *p* = 0.073). Inflammatory indices were significantly associated with CSFP. Lower HALP score was strongly and inversely associated with CSFP (OR 0.953, 95% CI 0.935–0.972, *p* < 0.001). In contrast, higher NLR, PLR, SII, and SIRI were all positively associated with CSFP (all *p* < 0.05).

Multivariable logistic regression analyses were performed using a predefined modeling strategy to evaluate the independent associations between inflammatory indices and CSFP. As shown in Table 3, five separate models were constructed, each including a single inflammatory index and a common set of clinical covariates. Across all models, male sex and DM emerged as consistent and independent predictors of CSFP. Male sex was associated with approximately a twofold increase in the likelihood of CSFP in all models (adjusted ORs ranging from 2.04 to 2.13, all *p* < 0.05). Similarly, DM remained independently associated with CSFP, with adjusted ORs ranging from 2.48 to 2.87 across models (all *p* < 0.05). Age was not independently associated with CSFP in any of the multivariable models. Among lipid and inflammatory biomarkers, HDL cholesterol and CRP showed borderline associations with CSFP across all models, falling short of conventional statistical significance. When inflammatory indices were examined individually, the HALP score showed an independent inverse association with CSFP (adjusted OR 0.951, 95% CI 0.930–0.972, *p* < 0.001). In contrast, the NLR did not retain statistical significance after multivariable adjustment. PLR, SII, and SIRI remained independently associated with CSFP.

ROC analysis showed that the HALP score significantly discriminated patients with CSFP, with an AUC of 0.698 (95% CI: 0.632–0.763, *p* < 0.001). Based on the Youden index, an optimal HALP cutoff of ≤56.4 yielded a sensitivity of 58.2% and a specificity of 77.0%. At this threshold, the PPV and NPV were 70.7% and 65.1%, respectively.

**Figure 2:**
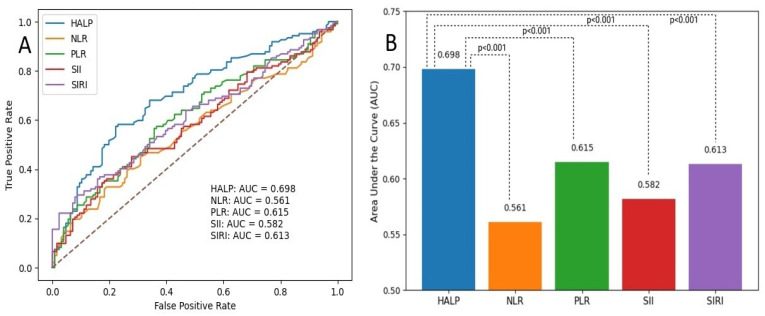
(**A**) Receiver operating characteristic curves of HALP score and inflammatory indices for predicting the coronary slow flow phenomenon (CSFP); (**B**) DeLong test comparison of areas under the curves (AUC) for HALP score and inflammatory indices in predicting the CSFP. Bar plots show the AUC values for HALP, NLR, PLR, SII, and SIRI. Pairwise comparisons between HALP and other inflammatory indices were performed using the DeLong test, demonstrating significantly higher discriminative performance of HALP (all *p* < 0.001). Abbreviations: HALP, Hemoglobin, Albumin, Lymphocyte, and Platelet score; NLR, Neutrophil-to-Lymphocyte Ratio; PLR, Platelet-to-Lymphocyte Ratio; SII, Systemic Immune Inflammation Index; SIRI, Systemic Immune Response Index.

The discriminative performance of the HALP score and other inflammatory indices for predicting the CSFP was evaluated using ROC curve analysis. As shown in Figure 2A, the HALP score demonstrated the highest diagnostic performance among the evaluated indices, with an AUC of 0.698. In comparison, the AUC values for NLR, PLR, SII, and SIRI were 0.561, 0.615, 0.582, and 0.613, respectively. A comparative analysis of AUC values is shown in Figure 2B. Pairwise comparisons using the DeLong test revealed that the AUC of the HALP score was significantly higher than that of all other inflammatory indices (all *p* < 0.001).

**Figure 3:**
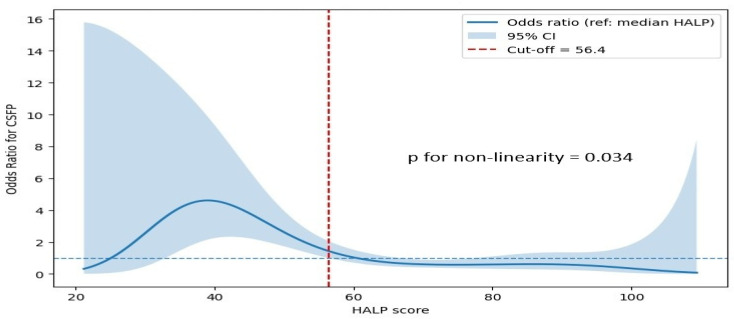
Restricted cubic spline analysis demonstrating the non-linear association between HALP score and risk of coronary slow flow phenomenon (CSFP). The solid line represents adjusted odds ratios for CSFP across the HALP score distribution, and the shaded area indicates the 95% confidence intervals (CIs). The reference odds ratio was set at 1.0. Abbreviations: HALP, Hemoglobin, Albumin, Lymphocyte, and Platelet score. The blue dotted horizontal line indicates the reference odds ratio of 1, corresponding to no increased or decreased risk.

The association between the HALP score and CSFP was further explored using RCS analysis. As illustrated in Figure 3, the HALP score demonstrated a significant non-linear relationship with the risk of CSFP (*p* for non-linearity = 0.034). Lower HALP values were associated with a steep increase in the odds of CSFP, whereas the risk progressively decreased with increasing HALP levels. A threshold effect was observed around a HALP value of approximately 56.4, beyond which the odds of CSFP approached or fell below unity. At higher HALP levels, the association plateaued, indicating a diminishing incremental protective effect.

**Figure 4:**
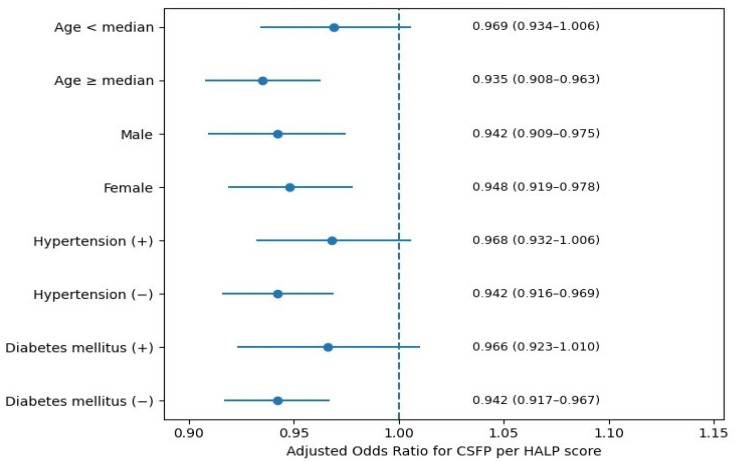
Subgroup analyses of the association between HALP score and the coronary slow flow phenomenon (CSFP). Forest plot showing adjusted odds ratios and 95% confidence intervals (CIs) for the association between HALP score and CSFP across predefined subgroups stratified by age, sex, hypertension, and diabetes mellitus. Abbreviations: HALP, Hemoglobin, Albumin, Lymphocyte, and Platelet score.

Subgroup analyses stratified by age, sex, hypertension, and DM demonstrated that the inverse association between the HALP score and the presence of the CSFP was consistent in direction across all evaluated subgroups (Figure 4). The association remained statistically significant in patients aged ≥ median age, in both male and female patients, and in those without hypertension or DM. In contrast, although the direction of the association was preserved, statistical significance was attenuated in patients aged < median age and in those with hypertension or DM. These findings likely reflect reduced statistical power in smaller subgroups rather than true effect modification.

## 4. Discussion

In this study, we demonstrated that the HALP score is independently associated with CSFP in patients with angina or ischemia and non-obstructive coronary arteries. Among the inflammation-related indices evaluated, the HALP score showed the strongest and most consistent association with CSFP across univariable and multivariable analyses, and superior discriminative performance in ROC analysis. Furthermore, the observed non-linear relationship between the HALP score and CSFP, characterized by a marked increase in risk at lower values, underscores the potential clinical relevance of immunonutritional status in this population. To the best of our knowledge, this is the first study to comprehensively evaluate the relationship between the HALP score and CSFP in patients with ANOCA/INOCA, extending current evidence on inflammatory biomarkers by integrating immune, nutritional, and hematologic components into a single predictive index.

Our findings can be contextualized within the known pathophysiology of CSFP. CSFP is not simply an incidental angiographic oddity but a condition characterized by diffuse microvascular and endothelial dysfunction [16]. Endothelial impairment plays a central role: CSFP patients often exhibit blunted endothelium-dependent vasodilation and reduced nitric oxide bioavailability. For example, flow-mediated dilation is significantly impaired in CSFP, partly attributable to elevated homocysteine levels, and nitric oxide levels are lower than normal, reflecting endothelial dysfunction [17]. Structurally, small intramural coronary arterioles in CSFP exhibit thickened walls and luminal narrowing, consistent with a microvascular disease process that increases flow resistance. This heightened microvascular tone and structural remodeling can explain the delayed contrast opacification seen angiographically in slow flow [18]. In parallel, there is abundant evidence of chronic inflammation in CSFP patients. Li et al. reported elevated levels of high-sensitivity C-reactive protein and interleukin-6 in CSFP [19]. These inflammatory mediators not only damage the endothelium but also promote oxidative stress and vasomotor dysfunction. Oxidative stress is now recognized as a contributor to microvascular angina and slow flow; excessive reactive oxygen species can scavenge nitric oxide, leading to vasoconstriction, endothelial injury, and microcirculatory spasm [20]. Taken together, CSFP is considered a diffuse, systemic small-vessel disorder characterized by endothelial dysfunction, inflammation, platelet hyperactivity, and oxidative damage. It is therefore intuitive that an index capturing several of these pathological domains—as the HALP score does—would be associated with CSFP.

Each component of the HALP score has a plausible mechanistic link to CSFP. Hemoglobin and albumin represent the patient’s oxygen-carrying capacity and nutritional or inflammatory status [21]. Lower hemoglobin (as reflected by a low HALP) may exacerbate myocardial ischemia in CSFP by reducing oxygen delivery to the myocardium; it can also indicate anemia of chronic disease or general ill health associated with inflammation [22]. Albumin, a negative acute-phase reactant, declines in the setting of systemic inflammation and oxidative stress; hypoalbuminemia also signifies poor nutrition and has been linked to endothelial dysfunction through loss of its antioxidant and colloid osmotic functions [23,24]. A low albumin level (and thus a lower HALP) in CSFP patients likely reflects chronic inflammation and an increased oxidative stress burden. Lymphocyte count is a sensitive gauge of immune status: stress and inflammation tend to cause relative lymphopenia as neutrophils and monocytes expand [25]. CSFP patients have been shown to harbor heightened inflammation—for instance, the NLR is significantly elevated in CSFP, which corresponds to lower lymphocyte counts [26]. Thus, the lymphocyte component of HALP will be reduced in pro-inflammatory states, such as CSFP. Platelets, conversely, are often elevated or hyper-reactive during systemic inflammation [27]. An increased platelet count (lowering HALP’s denominator) and heightened platelet activity can promote microvascular problems by forming microthrombi and releasing vasoactive substances. Indeed, CSFP individuals frequently exhibit platelet activation: mean platelet volume is higher in CSFP than in controls, and elevated MPV is an independent predictor of the CSFP [28]. Larger, more reactive platelets can occlude microvessels or trigger spasm, linking thrombocytosis and platelet activity to CSFP pathogenesis. In summary, a low HALP score (anemia, hypoalbuminemia, lymphopenia, and thrombocytosis) serves as a composite biomarker of the same processes—malnutrition, inflammation, endothelial perturbation, and platelet activation—that are thought to underlie CSFP. This biological coherence strengthens the plausibility of our observed association between HALP and CSFP.

The relationship between HALP and CSFP that we observed aligns with and extends prior studies on inflammatory indices in cardiovascular disease. HALP has been studied in ACS for its prognostic implications for microvascular disease. Toprak et al. reported that ST-elevation myocardial infarction patients with low HALP scores were far more likely to develop the no-reflow phenomenon after primary PCI, a complication of impaired microvascular reperfusion [29]. This finding is highly pertinent because the no-reflow phenomenon shares pathophysiological features with CSFP—both involve microvascular dysfunction and inflammatory injury leading to reduced perfusion [30]. The fact that HALP predicts no-reflow lends credence to our observation that HALP is low in primary CSFP; in both scenarios, an adverse systemic milieu predisposes to microvascular flow abnormalities. HALP has also shown prognostic value in chronic cardiovascular disease. For example, a high HALP score was associated with lower long-term mortality in patients with HF [31], and in a recent cohort of atrial fibrillation patients with concomitant ACS, adding HALP significantly improved risk stratification for major adverse events [32]. These studies collectively underscore that HALP is a robust integrative marker of risk across a spectrum of cardiovascular conditions. Our work adds to this literature by suggesting that HALP is not only a prognostic indicator in overt ACS or HF, but may also serve as a marker of an insidious ischemic condition like CSFP.

We also compared HALP with other inflammatory biomarkers evaluated in CSFP and related disorders. Prior investigations have consistently shown that simple leukocyte-based indices are elevated in CSFP. Dogan et al. found the NLR significantly higher in CSFP and identified it as an independent predictor of CSFP [26]. Similarly, Oylumlu et al. demonstrated that the PLR was higher in CSFP and correlated with TFC, and that an elevated PLR was independently associated with CSFP [33]. More recently, composite inflammatory scores have been explored. Dai et al. reported that SII was significantly elevated in CSFP patients and was an independent predictor of CSFP [34]. Likewise, the SIRI was elevated in CSFP and increased progressively with the number of arteries involved; in a 2024 study, it was an independent predictor in multivariable analysis [11]. Our results with HALP are consistent: HALP can be viewed as another composite index, though unique for incorporating hemoglobin and albumin. While indices such as NLR and SII focus solely on inflammatory cell counts, the HALP score integrates the immune response with the patient’s nutritional and hematologic status. This broader scope may confer advantages to HALP. For instance, anemia and hypoalbuminemia are known risk markers in cardiovascular disease on their own [35,36], often reflecting chronic inflammation or frailty; HALP includes these dimensions, whereas NLR/PLR do not. In our comparative analysis, the HALP score demonstrated a stronger and more consistent association with CSFP than other evaluated inflammatory indices. In multivariable logistic regression models adjusted for key clinical covariates, HALP retained its independent inverse relationship with CSFP, whereas NLR lost statistical significance. Although PLR, SII, and SIRI remained significant predictors, their effect sizes were less robust than those of HALP. Moreover, ROC curve analysis revealed that HALP yielded the highest discriminative performance among all indices, significantly surpassing those of NLR, PLR, SII, and SIRI (all DeLong *p*-values < 0.001). These findings suggest that HALP, by integrating hematologic and nutritional-inflammatory components, may offer a more comprehensive reflection of the underlying pathophysiology of CSFP and could serve as a practical and informative marker in clinical assessment. Nonetheless, further studies are warranted to validate these results and to determine the additive value of HALP in broader clinical settings.

From a clinical perspective, the HALP score may serve as a simple screening tool for CSFP. Patients with CSFP often experience recurrent angina, ST-segment changes, or even episodes of ACS despite having no significant epicardial stenoses. This “microvascular angina” can impair quality of life and, in some cases, has prognostic implications, including arrhythmia or cardiomyopathy risk [37]. Yet clinicians currently lack quantitative markers to identify patients with CSFP. Our findings suggest that HALP could fill this gap. The score is derived from routine laboratory parameters and is thus readily available; a notably low HALP in a patient with angiographically confirmed CSFP may alert physicians to a high inflammatory burden and indicate the need for closer monitoring.

While our findings suggest that the HALP score is a promising biomarker for identifying patients at higher risk of CSFP, further studies are needed to evaluate its role in long-term management and response to interventions. Despite notable advances in individualized care for patients with CCSs, significant gaps remain in optimizing risk stratification and therapeutic strategies, including the potential use of anti-inflammatory approaches [38]. While our study focused on the diagnostic association between HALP and CSFP, these insights underscore the importance of incorporating these markers in future studies examining long-term outcomes and responses to lifestyle interventions, such as physical activity or cardiac rehabilitation. Given its simplicity and accessibility, HALP may aid in the early identification of patients at higher risk for microvascular dysfunction, thereby facilitating more tailored monitoring and preventive strategies.

### Study Limitations and Strengths

This study has several limitations that should be acknowledged. First, its retrospective design inherently limits the ability to establish causal relationships and may introduce selection or information bias. Second, this was a single-center study conducted at a tertiary care hospital, which may limit the generalizability of the findings to broader populations or other clinical settings. Third, our analysis lacked longitudinal follow-up data, preventing evaluation of the HALP score’s predictive value for clinical outcomes or progression of CSFP over time. Moreover, although our sample size was adequate to detect statistical associations, it may not be sufficient to account for all potential confounding factors or to conduct more granular subgroup analyses. Finally, although we adjusted for several relevant covariates and compared HALP with multiple established inflammatory indices, residual confounding from unmeasured variables cannot be excluded.

Despite these limitations, the study has notable strengths. It is, to our knowledge, the first to systematically evaluate the HALP score relative to CSFP, integrating a wide array of inflammatory and immunonutritional indices within a single analytic framework. The use of standardized angiographic assessment, multivariable modeling with predefined covariates, and comparative ROC analysis all enhances the methodological rigor and clinical relevance of the findings. The HALP score is calculated from routine laboratory values, making it a practical, potentially scalable tool across diverse healthcare settings.

## 5. Conclusions

The present study identifies the HALP score as a promising, integrative biomarker independently associated with CSFP in patients with ANOCA/INOCA. By incorporating markers of inflammation, nutrition, and hematologic status, HALP provides a unique reflection of the complex pathophysiology underlying CSFP. Its superior discriminative performance relative to commonly used inflammatory indices underscores its potential utility in clinical risk stratification. Given its derivation from routine laboratory tests, the HALP score may be a practical tool for evaluating patients with anginal symptoms and normal coronary angiograms. Nonetheless, our findings warrant external validation through multicenter, prospective studies to establish its predictive value and generalizability across broader populations.

## Figures and Tables

**Table 1:** Baseline demographic, clinical, and laboratory characteristics of the groups.

	Patients with Coronary Slow Flow (n = 122)	Patients with Coronary Normal Flow (n = 126)	*p*-Value
Demographics and comorbidities			
Age (years)	57.0 ± 6.5	57.0 ± 7.2	0.926
Male sex, n (%)	51 (41.8)	73 (57.9)	0.011
Hypertension, n (%)	46 (37.7)	43 (34.1)	0.557
Diabetes mellitus, n (%)	33 (27.0)	22 (17.5)	0.069
Smoking, n (%)	29 (23.8)	34 (27.0)	0.561
Drug usage on admission, n (%)			
β-blocker	19 (15.6)	25 (19.8)	0.395
ACE inhibitor	23 (18.9)	23 (18.3)	0.904
ARB	20 (16.4)	15 (11.9)	0.310
Statin	16 (13.1)	24 (19.4)	0.204
Laboratory findings			
White blood cells (×10^3^/mm^3^)	8.1 ± 2.1	8.4 ± 2.4	0.290
Hemoglobin (g/dL)	14.1 ± 0.7	13.8 ± 0.8	0.004
Platelets (×10^3^/mm^3^)	294.6 ± 33.0	276.0 ± 31.8	<0.001
Neutrophils (×10^3^/mm^3^)	6.2 ± 1.1	5.9 ± 1.1	0.176
Lymphocytes (×10^3^/mm^3^)	2.9 ± 0.5	3.0 ± 0.5	0.167
Monocytes (×10^3^/mm^3^)	0.9 ± 0.2	0.8 ± 0.1	<0.001
Serum creatinine (mg/dL)	1.0 ± 0.6	1.0 ± 0.7	0.591
Serum albumin (mg/dL)	41.1 ± 2.2	43.0 ± 1.9	<0.001
AST (U/L)	23.6 ± 7.4	25.5 ± 31.5	0.516
ALT (U/L)	23.0 ± 9.2	23.1 ± 12.8	0.932
Total cholesterol (mg/dL)	179.2 ± 31.3	188.5 ± 39.4	0.081
Triglyceride (mg/dL)	186.2 ± 61.0	180.3 ± 60.8	0.475
HDL-cholesterol (mg/dL)	40.3 ± 5.6	41.7 ± 5.9	0.069
C-reactive protein (mg/L)	8.3 ± 9.3	4.7 ± 5.2	0.030
Inflammatory indices			
HALP score	56.16 ± 14.07	65.89 ± 14.56	<0.001
Neutrophil-to-lymphocyte ratio	2.14 ± 0.60	2.00 ± 0.45	0.046
Platelet-to-lymphocyte ratio	10.44 ± 2.48	9.57 ± 2.19	0.004
Systemic immune inflammation index	612.38 ± 192.18	554.29 ± 144.65	0.008
Systemic inflammation response index	19.52 ± 6.89	16.56 ± 4.39	<0.001
TIMI frame count			
LAD	59.7 ± 21.5	22.5 ± 4.6	<0.001
RCA	59.1 ± 23.8	17.2 ± 3.1	<0.001
LCx	61.6 ± 21.1	17.8 ± 3.7	<0.001
Mean TFC	60.1 ± 11.4	19.2 ± 2.2	<0.001

Abbreviations: ALT, Alanine Aminotransferase; ARB, Angiotensin Receptor Blocker; AST, Aspartate Aminotransferase; HALP, Hemoglobin, Albumin, Lymphocyte, and Platelet score; HDL, High-Density Lipoprotein; LAD, Left Anterior Descending Artery; LCx, Circumflex Artery; RCA, Right Coronary Artery; TFC, Thrombolysis in Myocardial Infarction Frame Count.

**Table 2:** Univariable logistic regression analysis of clinical and laboratory parameters associated with the coronary slow flow phenomenon.

Variable	Odds Ratio	95% Confidence Interval	*p*-Value
Age	0.998	0.962–1.035	0.926
Male sex	1.917	1.158–3.176	0.011
Hypertension	1.168	0.695–1.964	0.557
Diabetes mellitus	1.753	0.953–3.223	0.071
Total cholesterol	0.994	0.986–1.002	0.116
HDL-cholesterol	0.957	0.912–1.004	0.073
C-reactive protein	1.085	1.019–1.156	0.011
HALP score	0.953	0.935–0.972	<0.001
NLR	1.624	1.007–2.620	0.047
PLR	1.180	1.051–1.325	0.005
SII	1.002	1.001–1.004	0.009
SIRI	1.095	1.045–1.148	<0.001

Abbreviations: HALP, Hemoglobin, Albumin, Lymphocyte, and Platelet score; HDL, High-Density Lipoprotein; NLR, Neutrophil-to-Lymphocyte Ratio; PLR, Platelet-to-Lymphocyte Ratio; SII, Systemic Immune Inflammation Index; SIRI, Systemic Immune Response Index.

**Table 3:** Multivariable logistic regression models comparing HALP score and other inflammatory indices for the prediction of the coronary slow flow phenomenon.

Variable	Model 1 ^ⴕ^ (HALP)	Model 2 ^ⴕ^ (NLR)	Model 3 ^ⴕ^ (PLR)	Model 4 ^ⴕ^ (SII)	Model 5 ^ⴕ^ (SIRI)
Age	1.013 (0.964–1.064), *p* = 0.614	1.009 (0.964–1.057), *p* = 0.696	1.012 (0.966–1.061), *p* = 0.604	1.010 (0.964–1.058), *p* = 0.688	1.005 (0.958–1.054), *p* = 0.854
Male sex	2.110 (1.168–3.813), *p* = 0.013	2.078 (1.183–3.652), *p* = 0.011	2.127 (1.205–3.757), *p* = 0.009	2.047 (1.161–3.609), *p* = 0.013	2.037 (1.145–3.622), *p* = 0.015
Diabetes mellitus	2.870 (1.278–6.450), *p* = 0.011	2.478 (1.173–5.232), *p* = 0.017	2.549 (1.190–5.460), *p* = 0.016	2.599 (1.223–5.523), *p* = 0.013	2.578 (1.200–5.539), *p* = 0.015
HDL-cholesterol	0.948 (0.896–1.002), *p* = 0.060	0.954 (0.907–1.004), *p* = 0.072	0.951 (0.903–1.002), *p* = 0.061	0.954 (0.906–1.004), *p* = 0.071	0.953 (0.904–1.004), *p* = 0.069
C-reactive protein	1.058 (0.992–1.129), *p* = 0.085	1.059 (0.996–1.126), *p* = 0.066	1.059 (0.995–1.127), *p* = 0.071	1.059 (0.996–1.126), *p* = 0.068	1.060 (0.995–1.130), *p* = 0.072
HALP score	0.951 (0.930–0.972), *p* < 0.001	—	—	—	—
NLR	—	1.589 (0.940–2.687), *p* = 0.084	—	—	—
PLR	—	—	1.191 (1.045–1.357), *p* = 0.009	—	—
SII	—	—	—	1.002 (1.000–1.004), *p* = 0.017	—
SIRI	—	—	—	—	1.098 (1.041–1.158), *p* < 0.001

Abbreviations: HALP, Hemoglobin, Albumin, Lymphocyte, and Platelet score; HDL, High-Density Lipoprotein; NLR, Neutrophil-to-Lymphocyte Ratio; PLR, Platelet-to-Lymphocyte Ratio; SII, Systemic Immune Inflammation Index; SIRI, Systemic Immune Response Index. ^ⴕ^ All models were adjusted for age, sex, diabetes mellitus, HDL-cholesterol, and C-reactive protein. Values are presented as adjusted odds ratios with 95% confidence intervals.

## Data Availability

Data are available from the corresponding author upon reasonable request. The data are not publicly available due to privacy or ethical restrictions.

## Supplementary Materials

The following supporting information can be downloaded at: https://www.mdpi.com/article/10.3390/jcm15031302/s1, Video S1: Representative angiographic image demonstrating normal coronary flow in a patient with typical anginal symptoms and non-obstructive coronary arteries. The contrast dye progresses rapidly and smoothly through the epicardial coronary arteries, consistent with normal Thrombolysis in Myocardial Infarction (TIMI) 3 flow. Video S2: Representative angiographic image illustrating the coronary slow flow phenomenon (CSFP) in a patient with chest pain and angiographically non-obstructive coronary arteries. The contrast dye progresses sluggishly through the coronary vasculature, indicative of delayed distal vessel opacification characteristic of CSFP.
